# Supplementary material for: Highly variable iron content modulates iceberg-ocean fertilisation and potential carbon export
Source: Nat Commun. 2019 Nov 20;10:5261. doi: 10.1038/s41467-019-13231-0 (PMC6868171; doi:10.1038/s41467-019-13231-0)
Supplement: Supplementary file 1 — SUPPLEMENTARY INFORMATION [file 41467_2019_13231_MOESM1_ESM.pdf]

Supplementary Information for Highly variable iron content modulates iceberg-ocean fertilisation and potential carbon export by Hopwood et al.

Supplementary Note 1 Theoretical melt rates for icebergs in open ocean environments

We investigate the vertical structure of iceberg melt using the line plume model of Ref[1], which solves four equations that conserve mass, momentum, heat, and salt along a vertical, melting ice face. This model formulation was previously used in Ref[2] (e.g., see Supplementary Figure 8 therein) to show that ocean density stratification can impede iceberg melt upwelling. Initial conditions for temperature and salinity are prescribed from conductivity-temperature-depth (CTD) profiles from southeast Greenland and the North Weddell Sea. Temperature and salinity profiles were extracted from British Oceanographic Data Centre for the regions 59.5–62° N, 42–75° W (SE Greenland) and 59–64° S, 46–56° W (Antarctic Peninsula) to represent regions with contrasting stratification and ocean temperatures.

We force the model at the iceberg keel depth of 200 m with an initial freshwater input of 10^{-7} m^2 s^{-1}. Due to the density stratification, melt-driven plumes quick reach neural density (i.e., vertical velocity tends to zero), which generates a series of stacked melt-driven convection cells along the ice face (Supplementary Figure 1). These convective cells generally become larger as water column temperature increases, resulting in larger convective cells for the Greenland scenario (Supplementary Figure 1, left) compared to the Antarctic Peninsula scenario (Supplementary Figure 1, right).

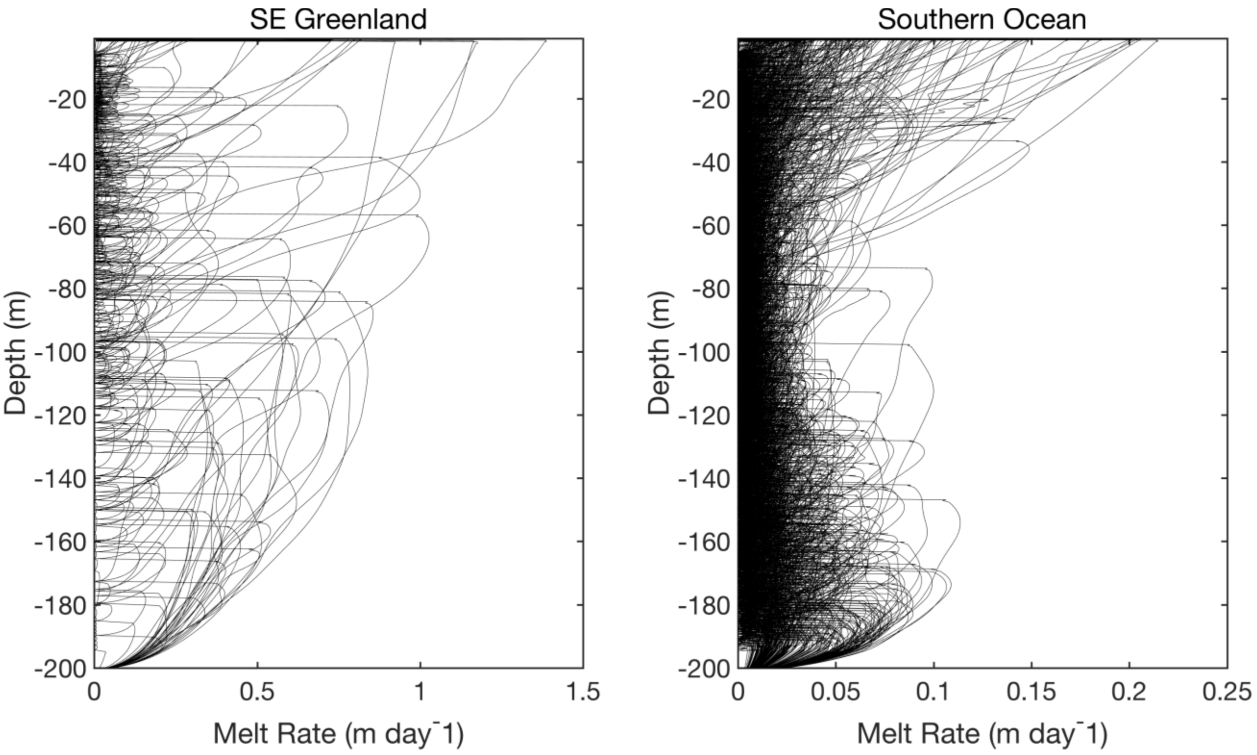

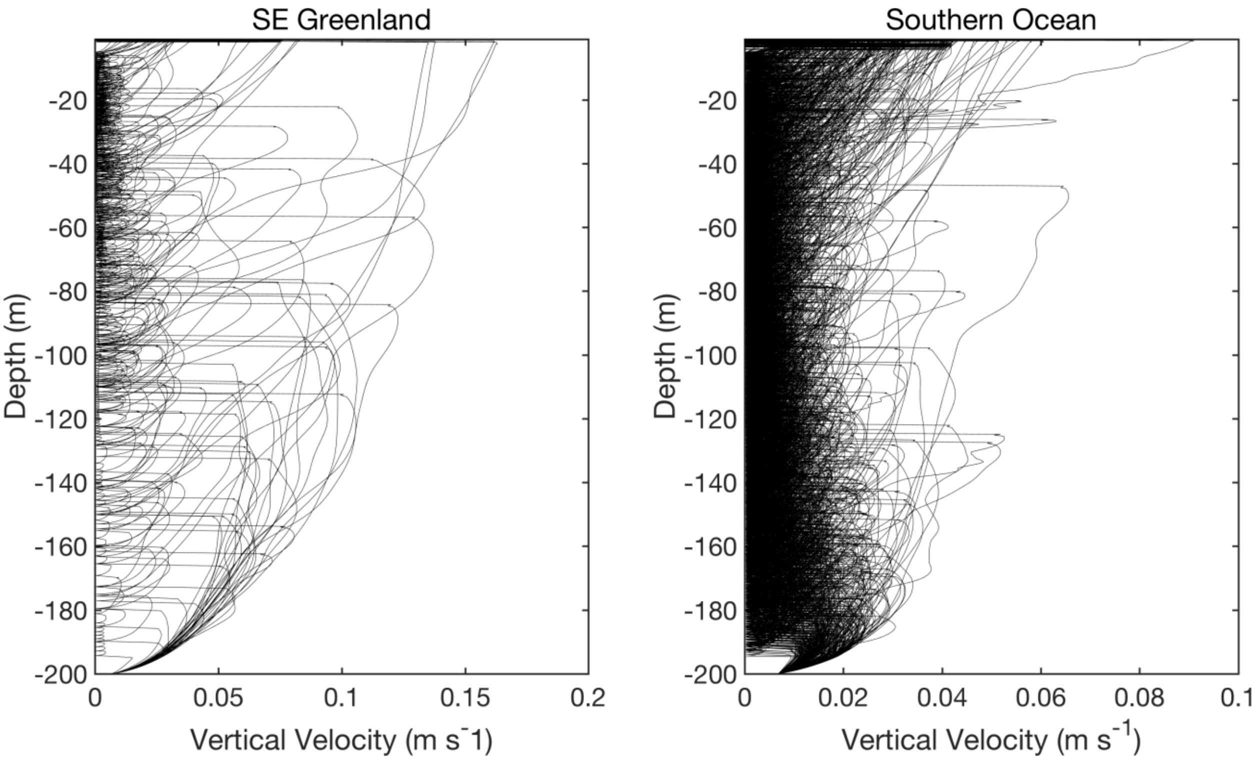

Supplementary Figure 1: Modeled iceberg melt rate (top) and plume vertical velocity (bottom) for a 200 m draft iceberg in a region south of Greenland (left), or east of the Antarctic Peninsula (right).

Supplementary Note 2 Comparison of TdFe between regions and catchments

All samples are grouped by 'catchment' (the local geographical area over which the samples were collected, often spanning multiple glacier systems and hydrological catchments). Catchments with at least 8 datapoints (Fildes, Yelcho, E Greenland, Godthåbsfjord, Upernarvik, Uummannaq, Jökulsárlón, Pia and Kongsfjorden) were then subject to analysis of variance adjusted for multiple pairwise comparisons (i.e. Tukey Test). Catchments were then grouped into broader geographical regions (Antarctica, Greenland, Iceland, Patagonia and Svalbard) and re-analysed.

	Fildes	Upernarvik	Yelcho	Kongsfjorden	Godthåbsfjord	E Greenland	Pia	Uummannaq
Jökulsárlón	<0.001	0.003	<0.001	<0.001	<0.001	<0.001	<0.001	<0.001
Fildes		1.000	0.993	0.965	0.975	0.983	0.993	0.998
Upernarvik			1.000	1.000	1.000	1.000	1.000	1.000
Yelcho				1.000	1.000	1.000	1.000	1.000
Kongsfjorden					1.000	1.000	1.000	1.000
Godthåbsfjord						1.000	1.000	1.000
E Greenland							1.000	1.000
Pia								1.000

Supplementary Table 1: P values for Fe data sorted by catchment subject to a pairwise multiple comparison test (Tukey Test).

	Iceland	Antarctica	Greenland	Svalbard
Patagonia	<0.001	0.978	1.000	1.000
Svalbard	<0.001	0.934	1.000	
Greenland	<0.001	0.934		
Antarctica	<0.001			

Supplementary Table 2: P values for Fe data sorted by geographic region subject to a pairwise multiple comparison test (Tukey Test).

Acknowledgements

Temperature/salinity data to construct Supplementary Figure 1 was extracted from historical CTD profiles, provided by the British Oceanographic Data Centre.

Supplementary References

1. Jenkins, A. Convection-Driven Melting near the Grounding Lines of Ice Shelves and Tidewater Glaciers. *J. Phys. Oceanogr.* **41**, 2279–2294 (2011).

2. Moon, T. *et al.* Subsurface iceberg melt key to Greenland fjord freshwater budget. *Nat. Geosci.* **11**, 49–54 (2018).
